# Modulation of β-amyloid aggregation by graphene quantum dots

**DOI:** 10.1098/rsos.190271

**Published:** 2019-06-26

**Authors:** Changliang Liu, Huan Huang, Lilusi Ma, Xiaocui Fang, Chen Wang, Yanlian Yang

**Affiliations:** 1 CAS Key Laboratory of Standardization and Measurement for Nanotechnology, CAS Key Laboratory of Biological Effects of Nanomaterials and Nanosafety, CAS Center for Excellence in Nanoscience, National Center for Nanoscience and Technology, Beijing 100190, People's Republic of China; 2 University of Chinese Academy of Sciences, Beijing 100049, People's Republic of China

**Keywords:** graphene quantum dots, β-amyloid aggregation, modulation

## Abstract

Misfolding and abnormal aggregation of β-amyloid peptide is associated with the onset and progress of Alzheimer's disease (AD). Therefore, modulating β-amyloid aggregation is critical for the treatment of AD. Herein, we studied the regulatory effects and mechanism of graphene quantum dots (GQDs) on 1–42 β-amyloid (Aβ_1–42_) aggregation. GQDs displayed significant regulatory effects on the aggregation of Aβ_1–42_ peptide as detected by thioflavin T (ThT) assay. Then, the changes of confirmations and structures induced by GQDs on the Aβ_1–42_ aggregation were monitored by circular dichroism (CD), dynamic light scattering (DLS) and transmission electron microscope (TEM). The *in vitro* cytotoxicity experiments further demonstrated the feasibility of GQDs on the regulation of Aβ_1–42_ aggregation. Meanwhile, the structural changes of a Aβ_1–42_/GQDs mixture in different pH revealed that electrostatic interaction was the major driving force in the co-assembly process of Aβ_1–42_ and GQDs. The proposed mechanism of the regulatory effects of GQDs on the Aβ_1–42_ aggregation was also deduced reasonably. This work not only demonstrated the potential feasibility of GQDs as therapeutic drug for AD but also clarified the regulatory mechanism of GQDs on the Aβ_1–42_ aggregation.

## 1. Introduction

Misfolding and abnormal aggregation of amyloid peptides or proteins is associated with a large number of diseases, such as Alzheimer's disease (AD), Parkinson's disease (PD), Huntington's disease, type II diabetes, mad cow disease and some types of cancers [1–3]. Among which, AD is the most common neurodegenerative disease which is characterized by the cerebral extracellular amyloid plaques [4,5]. The amyloid plaques are considered to be formed by the aggregation of β-amyloid peptide, which is an amphipathic polypeptide composed of 40–42 amino acids in humans [6]. Besides, Aβ_1–42_ is the major variant in cerebrospinal fluid (CSF), and its aggregation is the main component of the amyloid plaques. Therefore, Aβ_1–42_ peptide is considered as a major diagnostic biomarker and therapeutic target in the diagnosis and therapy of AD.

The sequence of Aβ_1–42_ peptide has been determined to be ‘AIVVGGVMLGIIAGKNSGVD EAFFVLKQHHVEYGSDHRFEAD’ in humans, which contains some hydrophobic amino acid residues and positive charge residues. These amino acids play a key role in the aggregation of Aβ_1–42_ driven by hydrophobic interaction, electrostatic interaction and hydrogen bond [7]. Aggregation of Aβ_1–42_ would form soluble oligomers and mature fibrils, which would damage the neurocytes and then induce the cerebral degeneration due to the neurotoxicity of the aggregates [8]. Thus, inhibiting or eliminating the formation of toxic Aβ_1–42_ aggregates would be an incredible strategy in AD therapy.

Recently, great efforts have been made to find efficient modulators to inhibit the toxic intermediates in the fibrillation process of amyloid peptides. Nanomaterials, including gold nanoparticles [9], inorganic semiconductors [10], polymer nanoparticles [11], magnetic nanoparticles [12], dendrimer [13] and carbon materials [14–22], have been intensely studied as new modulators in the field of amyloidosis with the development of nanotechnology. Notably, various carbon-based materials, such as graphene oxide (GO), carbon quantum dots and graphene quantum dots (GQDs), have drawn extensive attention due to their unique properties, versatile functionalities and good biocompatibility [23]. In particular, GQDs, as a zero-dimensional nanomaterial, possess superior chemical inertness, low toxicity, high fluorescent activity and excellent photostability [24]. Moreover, the unique structure and chemical properties make GQDs a research hotspot in the fields of imaging and sensing [25,26]. Several studies have reported that GQDs could be used to inhibit the aggregation of amyloid peptide [16–22], which provides the potentiality of GQDs in modulating amyloidogenesis. However, more characterization and studies were also needed to better understand the regulatory mechanism of GQDs for further application.

In this work, we investigated the modulation effects of GQDs on the aggregation of Aβ_1–42_. Results indicated that the regulatory behaviour of GQDs was mass ratio dependent and the critical mass ratio of Aβ_1–42_: GQDs was 1 : 1. Above the critical mass ratio, GQDs could protect Aβ_1–42_ peptide from aggregating into mature fibrils, instead of forming a hybrid network structure with β-turn confirmation with nontoxicity to cells according to the *in vitro* experiment. Then, the driven force of GQDs in the process of Aβ_1–42_ aggregation was studied, and results indicated that the electrostatic interaction between GQDs and Aβ_1–42_ peptide played an important role in the regulatory process. The regulation mechanism was further speculated according to the results in this work.

## 2. Material and methods

### 2.1. Materials

Aβ_1–42_ peptide was synthesized by Science Peptide Biological Technology Co., Ltd. (Shanghai, China). Graphite powder (size less than 30 µm) and 1,1,1,3,3,3-hexafluoro-2-propanol (HFIP) were purchased from Sinopharm Chemical Reagent Co., Ltd. Concentrated sulfuric acid, sodium nitrate, potassium permanganate, hydrochloric acid, hydrogen peroxide, sodium hydroxide and sodium carbonate were purchased from Beijing Chemical Plant. MTT (3-(4,5-dimethyl-2-thiazolyl)-2,5-diphenyl-2-H-tetrazolium bromide) was bought from Solarbio Science & Technology Co., Ltd. (Beijing, China). Deionized water used in all experiments was obtained from Milli-Q Integral 3 (Merck Millipore, France).

### 2.2. Preparation of graphene quantum dots

GQDs were prepared through two step reactions. First, GO was prepared from graphite powder according to the Hummer method with minor modification [27]. In the typical experiment, 4 g graphite powder and 3 g NaNO_3_ were dispersed into 150 ml concentrated H_2_SO_4_ under ice bath, then 18 g KMnO_4_ was slowly added into the mixture with vigorous vortex. Next, the mixture was removed from the ice bath and reacted for 120 h at room temperature. The obtained brownish grey paste was washed with 3% H_2_O_2_, 1% HCl and deionized water successively. The crude product was dialysed against deionized water for 2 days followed by ultrasonic exfoliating for 2 h to get well-dispersed GO.

The GQDs were prepared by incision of the prepared GO through oxidation reaction [28]. Briefly, the GO was diluted into 5 mg ml^−1^ with deionized water. Later, 30 ml GO solution, 1 ml concentrated H_2_SO_4_ and 1 ml HNO_3_ were mixed together and then put into a microwave reactor for 2 h at 200°C with the power of 500 W. Subsequently, the product was cooled to room temperature and neutralized with Na_2_CO_3_; the supernatant was centrifuged at 10 000 rpm for 30 min to remove big particles. Last, the solution was filtered through a 0.22 µm microporous membrane and further dialysed (molecular weight cut-off: 1000 Da) against water for 3 days to obtain the final brown GQDs products.

### 2.3. Characterization of GQDs

The morphology of GQDs was performed on an FEI Tecnai 20 transmission electron microscope (TEM, USA) at an acceleration voltage of 200 kV. The PL spectra of GQDs were recorded on an F-4600 fluorescence spectrometer (Hitachi, Japan) with the excitation wavelength of 360, 400, 440, 500 and 520 nm at the concentration of 1 mg ml^−1^. The emission spectra of GQDs with a series of concentrations were tested at the excitation wavelength of 360 nm. The structure of GQDs was further characterized by Raman spectra, Fourier-transform infrared spectra (FTIR) and X-ray photoelectron spectra (XPS). Specifically, Raman spectra were performed by irradiating laser at 514 nm in a Renishaw InVia Raman spectrometer (Renishaw plc, UK). FTIR was performed on a PerkinElmer FTIR spectrometer between 450 and 4000 cm^−1^, and the sample disc was made by tableting the mixture of GQDs and KBr before the test. XPS was investigated on an ESCALab220i-XL electron spectrometer (VG Scientific Ltd, UK) with a high-performance Al monochromatic source of 15 kV.

### 2.4. Pre-treatment of Aβ_1–42_ peptide

Aβ_1–42_ powder was dissolved in 1,1,1,3,3,3-hexafluoro-2-propanol (HFIP) at a concentration of 1 mg ml^−1^ and further incubated at 37°C for 6 h. The HFIP could be used as a hydrogen bond breaker to eliminate pre-existing homogeneity structures in the Aβ_1–42_. The Aβ_1–42_ solution was then separated into 100 µl per tube and stored at 4°C as stock solutions of Aβ_1–42_ peptide.

### 2.5. ThT assay

One hundred microlitre stock solution of Aβ_1–42_ peptide pre-treated as above was taken out and dried under nitrogen gas. The Aβ_1–42_ peptide was then re-dissolved into PBS buffer at a concentration of 50 µM with 10 µl DMSO as cosolvent. Thioflavin T (ThT) stock solution was prepared by dissolving ThT powder into PBS at a concentration of 1 mM and filtered by a 0.22 µm filter membrane before use. For each measurement, the incubated Aβ_1–42_ solutions at a concentration of 20 µM were prepared with ThT solution in phosphate buffer and GQDs at a mass ratio of 100 : 1, 10 : 1, 1 : 1 and 1 : 5. The final concentration of ThT was 10 µM, and free Aβ_1–42_ peptide was used as the control. The ThT fluorescence intensity was recorded every 10 min on a microplate reader (Tecan infinite M200, Switzerland) at excitation and emission wavelength of 450 nm and 482 nm, respectively.

### 2.6. Circular dichroism spectroscopy assay

To investigate the influences of GQDs in the aggregation process of Aβ_1–42_, changes of the secondary structure of Aβ_1–42_ with different amounts of GQDs were measured by CD analysis on a J-1500 spectropolarimeter (Jasco, Japan) at room temperature under a constant flow of nitrogen gas. Briefly, the Aβ_1–42_ peptides were dissolved in PB buffer (pH = 7.0) with 1% acetonitrile, then GQDs were added into the Aβ_1–42_ peptide solution with the mass ratio of Aβ_1–42_: GQDs in 100 : 1, 10 : 1, 1 : 1 and 1 : 5. Free Aβ_1–42_ peptide was prepared with the same steps mentioned before as control group. The samples were incubated in a shaker at 37°C before detection. Later, the samples were loaded into the CD cuvette to record CD spectra at 0 h, 2 h, 4 h, 6 h, 8 h and 10 h, respectively. GQDs solution with the same condition of samples was used as baseline in the experiment.

### 2.7. Cytotoxicity assay

Human neuroblastoma SH-SY5Y cells were cultured in Roswell Park Memorial Institute (RPMI)-1640 medium (Gibco, USA) supplemented with 10% fetal bovine serum (Gibco, USA) at 37°C in a humidified (5% CO_2_, 95% air) incubator. SH-SY5Y cells were seeded into 96-well microplates with a density of 8000 cells per well in 100 µl complete medium. After being incubated overnight at 37°C, the cells were treated with different media containing Aβ_1–42_ and Aβ_1–42_/GQDs (mass ratio = 1 : 1) mixtures. The concentration of Aβ_1–42_ was in the range of 5–100 µM. After being incubated for another 72 h at 37°C, 20 µl MTT (5 mg ml^−1^ of MTT in Hank's balancing buffer) was added into each well and the cells were incubated for another 4 h. Then, the medium was aspirated and 100 µl DMSO was added to dissolve the formazan salt. Cell viability was expressed as the percentage of viable cell relative to untreated group using the absorbance at 490 nm by an Infinite 200 microplate reader, TECAN. The cytotoxicity of GQDs was detected by the same procedure, and the concentration of GQDs was in the range of 22.5–1200 µg ml^−1^.

### 2.8. Dynamic light scattering assay

To detect the regulatory effect of GQDs in the process of Aβ_1–42_ aggregation, DLS tests were performed on a ZetasizerNano ZS nanoparticle size analyser (Malvern Instruments Ltd, UK). The Aβ_1–42_/GQDs mixture with the mass ratio of 1 : 1 was prepared in PBS buffer (pH = 7.0) first, and the final concentration of Aβ_1–42_ was 20 µM. After being incubated for 72 h at 37°C, the mixture was transferred into the DLS instrument to measure the size distribution of the peptides. Another sample was prepared with the same procedure mentioned above. At 72 h, the pH value of the mixture was adjusted to 10 by dropping NaOH solution (0.1 M) into the system. After 72 h incubation, the size distribution of the mixture was measured at the same condition.

The regulatory effect of GQDs for the Aβ_1–42_ fibrils was further investigated by the DLS assay. Aβ_1–42_ fibrils were made by dissolving the Aβ_1–42_ peptide into PBS (pH = 7.0) at a concentration of 20 µM and incubating for 72 h at 37°C. Then, the GQDs were added into the Aβ_1–42_ fibrils with the mass ratio of 1 : 1. DLS was performed to detect the changes in the size distribution of Aβ_1–42_ fibrils after the mixture being incubated for 72 h. Later, the value of pH was adjusted to 10 by 0.1 M NaOH solution and incubated for another 72 h, and the same procedure was performed to detect the size change of the Aβ_1–42_ fibrils.

### 2.9. Transmission electron microscope

First, the morphology of the Aβ_1–42_ aggregates with different contents of GQDs was determined by TEM. The samples were made by mixing the Aβ_1–42_ peptide and GQDs in PBS (pH = 7.0) with the mass ratio of 100 : 1 and 1 : 1 (Aβ_1–42_ : GQDs). After being incubated for 72 h at 37°C, the samples were dropped on the carbon-coated grid and observed on an Ht-7700 TEM (Hitachi, Japan) with the accelerate voltage of 80 kV. Then, we chose the mass ratio of 1 : 1 for Aβ_1–42_ : GQDs to investigate the regulatory effect of GQDs in the Aβ_1–42_ aggregation. The samples were prepared by incubating the Aβ_1–42_/GQDs mixture for 72 h before observation. Then, the pH value of the Aβ_1–42_/GQDs mixture was adjusted to 10 by NaOH solution (0.1 M) and reacting for another 72 h before observation by TEM. Additionally, the regulatory effect of GQDs for the mature Aβ_1–42_ fibrils was further investigated by TEM. Specifically, the samples were prepared by adding GQDs into the Aβ_1–42_ mature fibrils with the mass ratio of 1 : 1, and the TEM was executed after 72 h of incubation. Then, the pH value was adjusted to 10 and the mixture was incubated for another 72 h before the TEM assay.

## 3. Results and discussion

### 3.1. Preparation and characterization of GQDs

**Figure 1.**
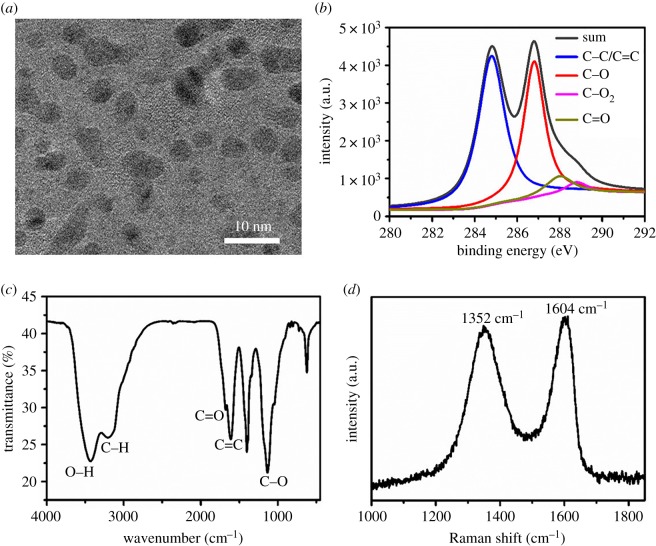
Morphology and spectroscopic characterizations of graphene quantum dots (GQDs). (*a*) TEM, scale bar is 10 nm; (*b*) XPS spectrum; (*c*) FTIR spectrum and (*d*) Raman spectrum.

GQDs were prepared through two steps. First, graphite powder was oxidized to GO according to the Hummer method [27]. Then, the GO was further oxidized to GQDs in the presence of concentrated H_2_SO_4_ and HNO_3_. The prepared GQDs exhibited a sheet-like structure and monodisperse in aqueous according to the TEM images (figure 1*a*). Size distribution of the GQDs was measured and the statistics were processed by Nano Measurer Software 1.2.0 according to the TEM results, which revealed the lateral size as 4.0 ± 0.7 nm (electronic supplementary material, figure S1). Fluorescent properties were further investigated by PL spectra with different excitation wavelengths as shown in electronic supplementary material, figure S2. Results demonstrated an excitation-dependent behaviour of the emission spectra with the strongest PL peak shifting to longer wavelength when the excitation wavelength changed from 360 to 520 nm. Meanwhile, the strongest PL peak was nearly 500 nm with the excitation wavelength between 360 and 440 nm, and the maximum excitation wavelength was 360 nm. The emission spectra of GQDs also indicated a concentration-dependent PL behaviour at the excitation of 360 nm, and the fluorescent intensity was linearly correlated with the concentration at the range of 5–50 µg ml^−1^ (electronic supplementary material, figure S3). The XPS (figure 1*b*) and FTIR (figure 1*c*) results demonstrated a high amount of oxygen element resulting from the formation of epoxide, hydroxyl, carbonyl and carboxyl in the oxidation process. The peaks of 1352 and 1604 cm^−1^ in the Raman spectrum were in accordance with the D-band and G-band, which indicated the similar structure of GQDs with GO. The characterizations of GQDs revealed that the prepared GQDs not only have a conjugated structure like graphene but also have abundant oxygen-containing functional groups, which might contribute to the interactions between GQDs and Aβ_1–42_ such as hydrophobic interaction, π–π stacking interaction, electrostatic interaction and hydrogen bond.

### 3.2. Regulatory effects of GQDs in the aggregation process of Aβ_1–42_

**Figure 2.**
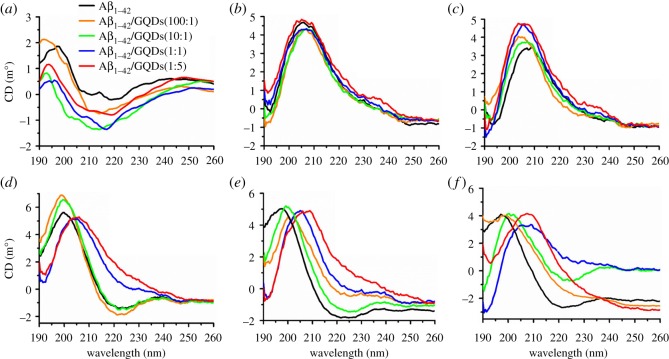
CD spectrum of Aβ_1–42_ peptide and Aβ_1–42_/GQDs mixture with different mass ratios (Aβ_1–42_ : GQDs = 100 : 1, 10 : 1, 1 : 1 and 5 : 1) at (*a*) 0 h, (*b*) 2 h, (*c*) 4 h, (*d*) 6 h, (*e*) 8 h and (*f*) 10 h.

As a material of regulator, how the GQDs interact with Aβ_1–42_ peptide and what changes it brings to the aggregation process of Aβ_1–42_ peptide are crucial questions. In this work, ThT assay, a widely used method for monitoring amyloid aggregation, was first performed to detect the aggregation kinetic process of Aβ_1–42_ peptide in the presence of GQDs. The ThT molecules could fluoresce upon binding to the β-sheet structure specifically, which provides information on the nucleation and elongation phases of Aβ_1–42_ assembly [29]. As shown in electronic supplementary material, figure S4, Aβ_1–42_ peptide displayed a quasi-sigmoid binding curve characterized by a lag time of about 120 min, and about 600 min period of successively increasing ThT binding followed by a plateau after 600 min. The aggregation of Aβ_1–42_ peptide was significantly different in the presence of GQDs compared with the free Aβ_1–42_ peptide, indicating that GQDs could influence the aggregation of Aβ_1–42_ peptide. Furthermore, CD spectra were performed to investigate the confirmational changes of Aβ_1–42_ peptide on the secondary structure with or without GQDs. Figure 2 exhibited the variations of CD signals along with different amounts of GQDs at different time intervals. At 0 h, all the samples possessed the typical α-helix confirmation with a positive band nearly 195 nm and several weak negative bands between 205 and 225 nm (figure 2*a*). After incubating for 2 h (figure 2*b*), both Aβ_1–42_ peptide and Aβ_1–42_/GQDs mixtures exhibited strong positive band at about 205 nm, which implied that the confirmation of Aβ_1–42_ was changed from α-helix to β-turn. This secondary structure could maintain for 4 h (figure 2*c*). While after another 2 h of incubation (figure 2*d*), obvious changes occurred in some of these groups. Pure Aβ_1–42_ peptide and Aβ_1–42_/GQDs mixtures that have low content of GQDs (Aβ_1–42_ : GQDs = 100 : 1 and 10 : 1) changed their confirmation to the typical β-sheet state with a positive band at 200 nm and a negative band at 222 nm. However, the Aβ_1–42_/GQDs mixtures that had higher GQDs content (Aβ_1–42_ : GQDs = 1 : 1 and 1 : 5) still remained in the β-turn states (figure 2*e*,*f*). Moreover, longer incubating time had little influence on the confirmations of pure Aβ_1–42_ peptide and low GQDs containing mixtures. Additionally, the confirmation of the Aβ_1–42_/GQDs mixture with the mass ratio of 1 : 1 and 1 : 5 showed little changes when the confirmation became to β-turn with the lapse of time.

**Figure 3.**
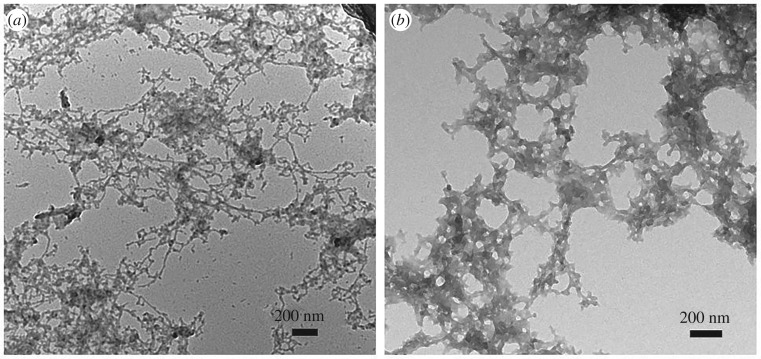
TEM images of the Aβ_1–42_ peptide/GQDs mixture with different mass ratios: (*a*) Aβ_1–42_ : GQDs = 100 : 1 and (*b*) Aβ_1–42_ : GQDs = 1 : 1.

To investigate the influences of GQDs in the process of Aβ_1–42_ aggregation, the morphologies of the Aβ_1–42_/GQDs mixture with low (Aβ_1–42_ : GQDs = 100 : 1) and high (Aβ_1–42_ : GQDs = 1 : 1) content of GQDs were performed by TEM and the results revealed significant differences as shown in figure 3. With low content of GQDs, the mixture presented the distinct typical fibrils structure (figure 3*a*), while incubated with high content of GQDs, the typical fibrils disappeared, instead forming a new hybrid network structure (figure 3*b*). Based on CD results, this typical hybrid network structure should also be different from typical fibrils in the secondary structure by containing little β-sheet. CD spectra give us the information that the critical mass ratio of GQDs and Aβ_1–42_ peptide was 1 : 1. Lower content of GQDs had little effect on the confirmation changes of Aβ_1–42_ peptide. If the content of GQDs was enough, GQDs could regulate the confirmation of Aβ_1–42_ peptide into the β-turn state and prevent the confirmation further transforming to the β-sheet state.

### 3.3. GQDs reduced the cytotoxicity induced by Aβ_1–42_

**Figure 4.**
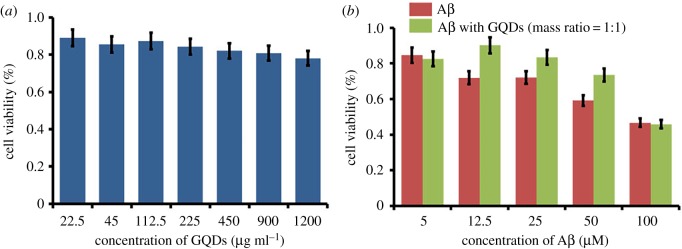
(*a*) Cytotoxicity of GQDs and (*b*) cytotoxicity of Aβ_1–42_ peptide and Aβ_1–42_/GQDs mixture with the mass ratio of 1 : 1.

In addition, the cytotoxicity of GQDs and Aβ_1–42_/GQDs mixture against SH-SY5Y cells was evaluated by MTT assay to investigate the safety of GQDs and the changes that GQDs brought to Aβ_1–42_ peptide in biology aspect. MTT assay based on succinic dehydrogenase activity in mitochondria was used to investigate the cell viabilities of SH-SY5Y cells in the presence of GQDs or Aβ_1–42_/GQDs mixture. GQDs were demonstrated to be a biocompatible material with slight toxicity to SH-SY5Y cells as shown in figure 4*a*. SH-SY5Y exhibited high cell viability up to 89% when the concentration of GQDs was 22.5 µg ml^−1^. Even 78% cells were still alive when the concentration increased to 1200 µg ml^−1^, which implied that GQDs could keep a relatively good biocompatibility from low to high dosage. What is more, the toxicity of Aβ_1–42_ peptide to SH-SY5Y cells was greatly reduced in the presence of GQDs. With the addition of Aβ_1–42_ and GQDs mixture (1 : 1 mass ratio), the cell viabilities of SH-SY5Y were promoted significantly when the concentration of Aβ_1–42_ peptide was located in the range of 12.5–50 µM (figure 4*b*), which was associated with the previous reports [7]. However, when the peptide concentration was decreased to 5 µM or increased to 100 µM, the effect of GQDs was almost neglected due to the indistinct toxicity deriving from the slim concentration and the non-affected peptide at high concentration. The possible reason for GQDs to reduce the cell toxicity of Aβ_1–42_ peptide might owe to the structure changes of Aβ_1–42_ peptide induced by GQDs as discussed in CD results. Overall, the results in MTT assay provide an opportunity to apply GQDs in a biological system due to its native low cytotoxicity, detection capability and the abilities to change peptide toxicity.

### 3.4. Detection of the regulatory mechanism of GQDs in the Aβ_1–42_ aggregation

The regulatory mechanism of GQDs in the Aβ_1–42_ aggregation is another critical question. Therefore, a series of contrast experiments were performed to understand the modulation mechanism of GQDs in the process of Aβ_1–42_ aggregation.

**Figure 5.**
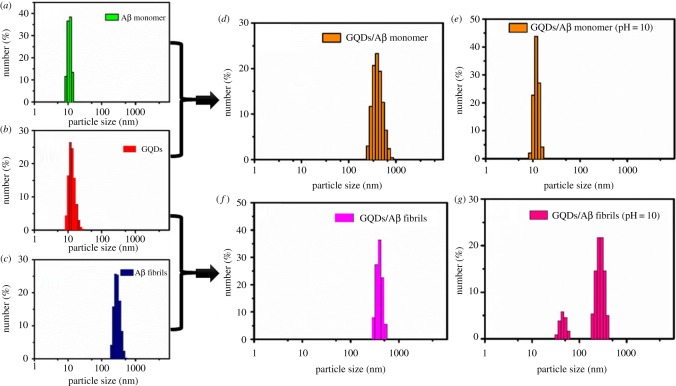
DLS spectra of (*a*) Aβ_1–42_ peptide, (*b*) GQDs, (*c*) Aβ_1–42_ fibrils, (*d*) Aβ_1–42_ peptide/GQDs with mass ratio of 1 : 1, (*e*) Aβ_1–42_ peptide/GQDs with mass ratio of 1 : 1 (pH = 10), (*f*) Aβ_1–42_ fibrils/GQDs with mass ratio of 1 : 1, (*g*) Aβ_1–42_ fibrils/GQDs with mass ratio of 1 : 1 (pH = 10).

DLS is a powerful tool to monitor particle size evolutions. In the experiment, native Aβ_1–42_ and Aβ_1–42_/GQDs mixtures were incubated at 37°C for 72 h to obtain Aβ_1–42_ fibrils and mature Aβ_1–42_/GQDs mixture. As shown in figure 5*a*,*b*, the size of GQDs or Aβ_1–42_ peptide ranged from 5–30 nm in DLS histograms. While the size distribution of the Aβ_1–42_/GQDs mixtures was strikingly grown to 100–900 nm, the particles with size less than 50 nm disappeared (figure 5*d*), indicating a co-assembly between GQDs and Aβ_1–42_ peptide. As in the previous report, the electrostatic interaction played an important role in the interaction between Aβ_1–42_ peptide and other electronegativity molecules [12]. The characterizations of GQDs demonstrated that GQDs possessed abundant oxygen-containing groups including carboxyl group, which is electronegative. Therefore, an increased pH value of above mixtures was administrated which could be used as a useful method to break the electrostatic interaction. As a result, the particle size of the mixture decreased greatly from 100–900 nm to 5–20 nm (figure 5*e*), which implied that a decomposition reaction occurred upon the pH jump. The resulting particle size was also close to the original size distribution of GQDs and free Aβ_1–42_ peptide; thus, the particles dispersed in this system should be the dissociative GQDs and Aβ_1–42_ peptide.

To further investigate whether the GQDs could disaggregate the mature Aβ_1–42_ fibrils, the size evolution of mature Aβ_1–42_ fibrils was also measured in the presence of GQDs. As the results shown in figure 5*c*, the particle size of Aβ_1–42_ fibrils was measured to be 150–550 nm and no obvious variations were observed after being mixed with GQDs. In addition, the size distribution of GQDs within 5–30 nm did not appear after adding the GQDs into the Aβ_1–42_ fibrils (figure 5*f*), which might result from the adsorption of GQDs on the Aβ_1–42_ fibrils. However, two distribution intervals appeared in histograms when the pH value was adjusted to 10 in figure 5*e*. The particle size distribution within 100–500 nm was consistent with the particle size of mature Aβ_1–42_ fibrils, indicating the negligible changes of the mature fibrils in the presence of GQDs upon the change of pH value. Besides, the size distribution within 20–60 nm was a little bigger than the free GQDs which might be due to the aggregation of GQDs with other components in the mixture, such as other GQDs formulation, unassembled peptide or dissociative oligomers.

**Figure 6.**
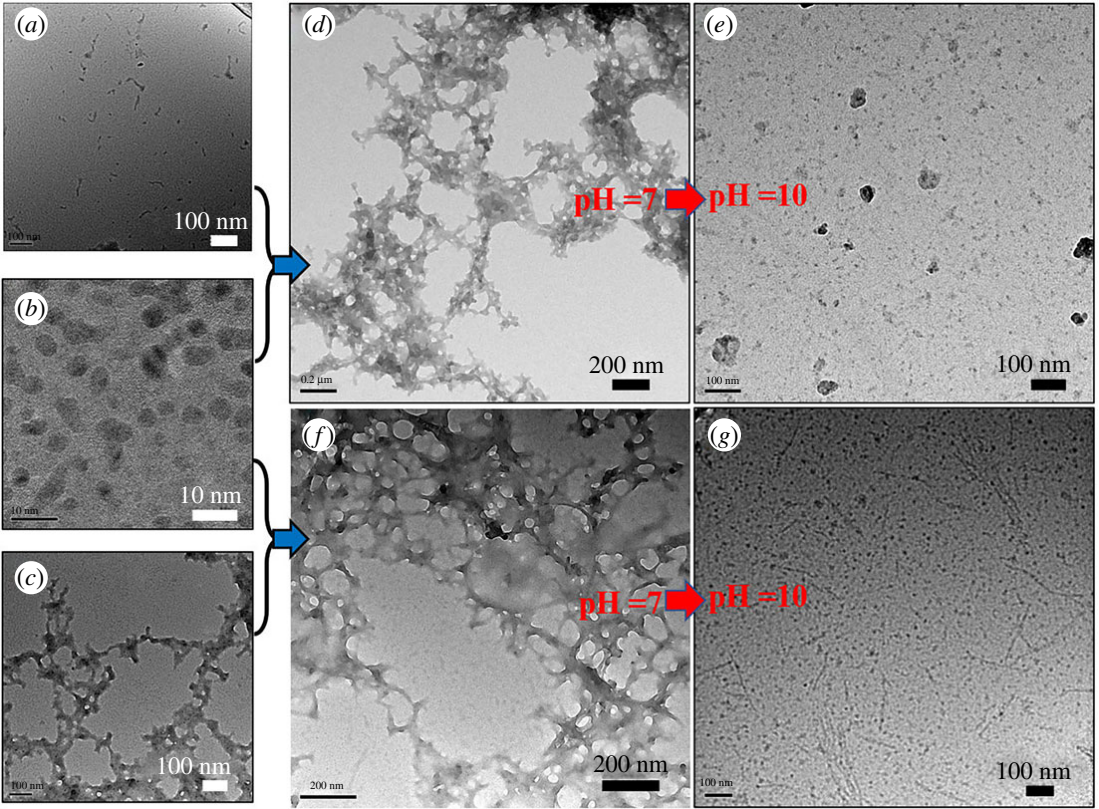
TEM images of (*a*) Aβ_1–42_ peptide; (*b*) GQDs; (*c*) Aβ_1–42_ fibrils; (*d*) Aβ_1–42_ peptide/GQDs with mass ratio of 1 : 1 in PBS (pH = 7); (*e*) Aβ_1–42_ peptide/GQDs with mass ratio of 1 : 1 in PBS buffer, the pH was adjusted to 10 by NaOH; (*f*) Aβ_1–42_ fibrils/GQDs with mass ratio of 1 : 1 in PBS (pH = 7); (*g*) Aβ_1–42_ fibrils/GQDs with mass ratio of 1 : 1 in PBS buffer, the pH was adjusted to 10 by NaOH.

Visual evidence was also provided by TEM images to confirm the morphology changes of the aggregates detected in DLS assay. As shown in the results, figure 6*a*–*c* exhibited the morphologies of native Aβ_1–42_ peptide, GQDs and fibrillar Aβ_1–42_, respectively. The rest of the images were the resulting morphologies after being treated with the same procedure as DLS tests. A distinct disordered network structure appeared when the mixture of the GQDs and Aβ_1–42_ peptide reacted for 72 h (figure 6*d*). While the pH value changed to 10, the cross-like structure disappeared as shown in figure 6*e*, instead of small particles scattering on the ground. In the image of GQDs and fibrillar Aβ_1–42_ mixture, only the cross-like structure was observed and the GQDs were not observed in the background (figure 6*f*). This result implied that the GQDs could absorb to the fibrillar Aβ_1–42_. When the pH value of the system was adjusted to 10, the mixture of GQDs and fibrillar Aβ_1–42_ displayed morphology with separated linear fibrils and small particles (figure 6*g*). All the morphology features shown in TEM images can be well coincident with the data in DLS.

Consequently, these results suggested that the mixture of GQDs and Aβ_1–42_ peptide could form aggregates with particle sizes of 100–900 nm. This assembly process was mainly driven by the electrostatic interaction, resulting in a unstable cross-like network structure, which would totally collapse when the environment slightly changed into alkaline. However, when the Aβ_1–42_ peptide assembled into mature fibrils, the GQDs could absorb on the fibrils, then the Aβ_1–42_ fibrils would maintain when the pH value jumped to 10. Thus, the network structure formed by GQDs and Aβ_1–42_ peptide should not contain the mature Aβ_1–42_ fibrils. The GQDs not only have the tendency to combine with Aβ_1–42_ peptide but also can prevent the conventional fibrosis process by this combination effect. It is worth mentioning that GQDs could not disintegrate the fibrillar Aβ_1–42_ that has already formed.

### 3.5. Proposed mechanism

**Figure 7.**
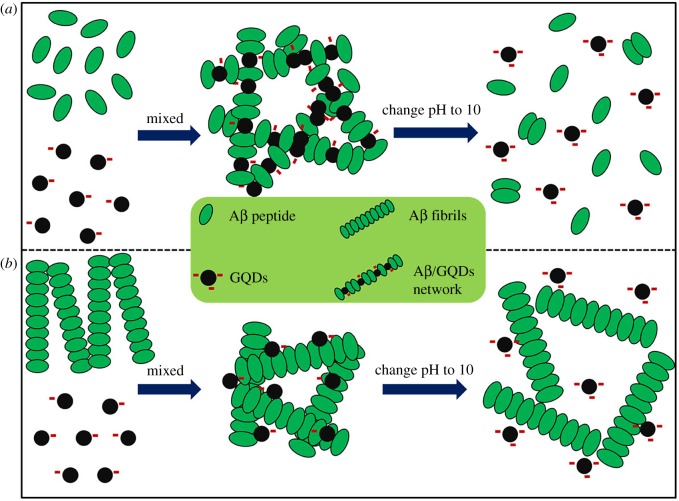
Proposed mechanistic diagrams for GQDs interact with Aβ_1–42_ peptide (*a*) and fibrils (*b*), and their structure changes after adjusting pH value to 10.

Based on the above analysis as well as CD spectra, DLS and TEM images, a possible mechanism was proposed in figure 7. If a sufficient quantity of GQDs was mixed with Aβ_1–42_ peptide (peptide/fibrils) and incubated for an extended period, the possible reaction processes were shown in figure 7. For the mixture of GQDs and Aβ_1–42_ peptide, they were prone to assembly into an unstable cross-like structure through electrostatic interaction, *π*–*π* stacking and hydrogen bonding, in which the electrostatic interaction is the main interaction. Because of the strong interaction between Aβ_1–42_ and GQDs, the secondary structure of the Aβ_1–42_ aggregates could maintain the β-turn state in the experimental period according to the results of CD spectra and cell cytotoxicity. Subsequent pH jump will raise the electronegativity of GQDs and Aβ_1–42_ peptide by the deprotonation of the carboxyl groups. As a result, electrostatic repulsive force dominated the interactions and impelled the disassembly of the Aβ_1–42_/GQDs co-assembled structure. For the mixture of GQDs and Aβ_1–42_ mature fibrils, the GQDs only absorbed on the surface of fibrils without damaging the performed fibrils. The adjustment of the pH value could only damage the absorption force between GQDs and Aβ_1–42_ fibrils but could not damage the fibrous structure.

## 4. Conclusion

According to the above research and discussions, the feasibility of using GQDs to regulate the aggregation of Aβ_1–42_ was demonstrated. The critical mass ratio of Aβ_1–42_ and GQDs was 1 : 1. What is more, GQDs could protect Aβ_1–42_ from being aggregated into big fibrils and maintain the aggregates in the nontoxic state but could not break the fibrils that had already formed. It is worth pointing out that the way that GQDs inhibited Aβ_1–42_ aggregation was forming a hybrid network structure by the strong interaction between GQDs and native Aβ_1–42_ peptide instead of keeping native Aβ_1–42_ freedom in solution. Additionally, great changes to the sizes and morphologies in the mixture upon pH jump strongly demonstrated the important role that the electrostatic interaction played in the interaction of GQDs and Aβ_1–42_. Moreover, *in vitro* tests produced credible evidence of the positive regulation of Aβ_1–42_ by GQDs. As a consequence, GQDs can be widely used as a functional nanomaterial in the area of protein amyloidogenesis.

## Supplementary Material

Characterizations of GQDs and the regulatory effect of GQDs on Aβ aggregation

Reviewer comments

## Data accessibility

The datasets supporting this article have been uploaded as part of the electronic supplementary material.

## References

[1] Eisenberg D, Jucker M 2012 The amyloid state of proteins in human diseases. Cell 148, 1188–1203. (10.1016/j.cell.2012.02.022) 22424229 PMC3353745

[2] Straub JE, Thirumalai D 2011 Toward a molecular theory of early and late events in monomer to amyloid fibril formation. Annu. Rev. Phys. Chem. 62, 437–463. (10.1146/annurev-physchem-032210-103526) 21219143 PMC11237996

[3] Uversky VN, Oldfield CJ, Dunker AK 2008 Intrinsically disordered proteins in human diseases: introducing the D2 concept. Annu. Rev. Biophys. 37, 215–246. (10.1146/annurev.biophys.37.032807.125924) 18573080

[4] Hardy J, Selkoe DJ 2002 The amyloid hypothesis of Alzheimer's disease: progress and problems on the road to therapeutics. Science 297, 353–356. (10.1126/science.1072994) 12130773

[5] Goedert M, Spillantini MG 2006 A century of Alzheimer's disease. Science 314, 777–781. (10.1126/science.1132814) 17082447

[6] Selkoe DJ 2001 Alzheimer's disease: genes, proteins, and therapy. Physiol. Rev. 81, 741–766. (10.1152/physrev.2001.81.2.741) 11274343

[7] Liu Y, Xu LP, Dai W, Dong H, Wen Y, Zhang X 2015 Graphene quantum dots for the inhibition of beta amyloid aggregation. Nanoscale 7, 19 060–19 065. (10.1039/C5NR06282A) 26515666

[8] Lansbury PT, Lashuel HA 2006 A century-old debate on protein aggregation and neurodegeneration enters the clinic. Nature 443, 774–779. (10.1038/nature05290) 17051203

[9] Shaw CP, Middleton DA, Volk M, Lévy R 2012 Amyloid-derived peptide forms self-assembled monolayers on gold nanoparticle with a curvature-dependent β-sheet structure. ACS Nano 6, 1416–1426. (10.1021/nn204214x) 22242947

[10] Yoo SI, Yang M, Brender JR, Subramanian V, Sun K, Joo NE, Jeong SH, Ramamoorthy A, Kotov NA 2011 Inhibition of amyloid peptide fibrillation by inorganic nanoparticles: functional similarities with proteins. Angew. Chem. Int. Edit. 50, 5110–5115. (10.1002/anie.201007824) PMC3516928 21495130

[11] Cabaleiro-Lago C, Quinlan-Pluck F, Lynch I, Lindman S, Minogue AM, Thulin E, Walsh DM, Dawson KA, Linse S 2008 Inhibition of amyloid β protein fibrillation by polymeric nanoparticles. J. Am. Chem. Soc. 130, 15 437–15 443. (10.1021/ja8041806) 18954050

[12] Mahmoudi M, Quinlan-Pluck F, Monopoli MP, Sheibani S, Vali H, Dawson KA, Lynch I 2013 Influence of the physiochemical properties of superparamagnetic iron oxide nanoparticles on amyloid beta protein fibrillation in solution. ACS Chem. Neurosci. 4, 475–485. (10.1021/cn300196n) 23509983 PMC3605812

[13] Gurzov EN et al. 2016 Inhibition of hIAPP amyloid aggregation and pancreatic beta-cell toxicity by OH-terminated PAMAM dendrimer. Small 12, 1615–1626. (10.1002/smll.201502317) 26808649

[14] Zhang M et al. 2013 The effect of graphene oxide on conformation change, aggregation and cytotoxicity of HIV-1 regulatory protein (Vpr). Biomaterials 34, 1383–1390. (10.1016/j.biomaterials.2012.10.067) 23153418

[15] Zhang M, Mao XB, Yu Y, Wang CX, Yang YL, Wang C 2013 Nanomaterials for reducing amyloid cytotoxicity. Adv. Mater. 25, 3780–3801. (10.1002/adma.201301210) 23722464

[16] Zeng HJ, Miao M, Liu Z, Yang R, Qu LB 2017 Effect of nitrogen-doped graphene quantum dots on the fibrillation of hen egg-white lysozyme. Int. J. Biol. Macromol. 95, 856–861. (10.1016/j.ijbiomac.2016.10.022) 27746357

[17] Yousaf M, Huang H, Li P, Wang C, Yang YL 2017 Fluorine functionalized graphene quantum dots as inhibitor against hIAPP amyloid aggregation. ACS Chem. Neurosci. 8, 1368–1377. (10.1021/acschemneuro.7b00015) 28230965

[18] Malishev R, Arad E, Bhunia SK, Shaham-Niv S, Kolusheva S, Gazit E, Jelinek R 2018 Chiral modulation of amyloid beta fibrillation and cytotoxicity by enantiomeric carbon dots. Chem. Commun. 54, 7762–7765. (10.1039/c8cc03235a) 29947369

[19] Liu Y, Xu LP, Wang Q, Yang B, Zhang X 2018 Synergistic inhibitory effect of GQDs-tramiprosate covalent binding on amyloid aggregation. ACS Chem. Neurosci. 9, 817–823. (10.1021/acschemneuro.7b00439) 29244487

[20] Chung YJ, Kim K, Lee BI, Park CB 2017 Carbon nanodot-sensitized modulation of Alzheimer's beta-amyloid self-assembly, disassembly, and toxicity. Small 13, 1700983 (10.1002/smll.201700983) 28714246

[21] Kim D et al. 2018 Graphene quantum dots prevent α-synucleinopathy in Parkinson's disease. Nat. Nanotechnol. 13, 812–818. (10.1038/s41565-018-0179-y) 29988049 PMC6351226

[22] Wang M et al. 2018 Graphene quantum dots against human IAPP aggregation and toxicity *in vivo*. Nanoscale 10, 19 995–20 006. (10.1039/C8NR07180B) PMC6212334 30350837

[23] Nurunnabi M, Khatun Z, Huh KM, Park SY, Lee DY, Cho KJ, Yk Lee 2013 *In vivo* biodistribution and toxicology of carboxylated graphene quantum dots. ACS Nano 7, 6858–6867. (10.1021/nn402043c) 23829293

[24] Ponomarenko LA, Schedin F, Katsnelson MI, Yang R, Hill EW, Novoselov KS, Geim AK 2008 Chaotic Dirac Billiard in graphene quantum dots. Science 320, 356–358. (10.1126/science.1154663) 18420930

[25] Zhu S et al. 2011 Strongly green-photoluminescent graphene quantum dots for bioimaging applications. Chem. Commun. 247, 6858–6860. (10.1039/C1CC11122A) 21584323

[26] Wang LJ et al. 2010 A graphene quantum dot with a single electron transistor as an integrated charge sensor. Appl. Phys. Lett. 97, 262113 (10.1063/1.3533021)

[27] Hummers WS, Offeman RE 1958 Preparation of graphitic oxide. J. Am. Chem. Soc. 80, 1339 (10.1021/ja01539a017)

[28] Sun Y, Wang S, Li C, Luo P, Tao L, Wei Y, Shi G 2013 Large scale preparation of graphene quantum dots from graphite with tunable fluorescence properties. Phys. Chem. Chem. Phys. 15, 9907–9913. (10.1039/C3CP50691F) 23673490

[29] Levine H 1993 Thioflavine-T interaction with synthetic Alzheimers-disease beta-amyloid peptides-detection of amyloid aggregation in solution. Protein Sci. 2, 404–410. (10.1002/pro.5560020312) 8453378 PMC2142377

