# Supplementary material for: Modulation of β-amyloid aggregation by graphene quantum dots
Source: R Soc Open Sci. 2019 Jun 26;6(6):190271. doi: 10.1098/rsos.190271 (PMC6599798; doi:10.1098/rsos.190271)
Supplement: Characterizations of GQDs and the regulatory effect of GQDs on Aβ aggregation [file rsos190271supp1.docx]

**Supporting Information**

**for**

**Modulation of β-Amyloid Aggregation by Graphene Quantum Dots**

Changliang Liu,^1, 2^ Huan Huang,^1, 2^ Lilusi Ma,^1^ Xiaocui Fang,^1^ Chen Wang,*^1, 2^ Yanlian Yang*^1, 2^

^1^CAS Key Laboratory of Standardization and Measurement for Nanotechnology, CAS Key Laboratory of Biological Effects of Nanomaterials and Nanosafety, CAS Center for Excellence in Nanoscience, National Center for Nanoscience and Technology, Beijing 100190, P. R. China.

^2^University of Chinese Academy of Sciences, Beijing 100049, P. R. China.

*Corresponding authors.

E-mail address: [yangyl@nanoctr.cn](mailto:yangyl@nanoctr.cn), wangch@nanoctr.cn.





**Figure S1.** Size distribution of the GQDs obtained by measuring the TEM images of GQDs using Nano Measurer Software 1.2.0. The average size is 4.0 ± 0.7 nm.


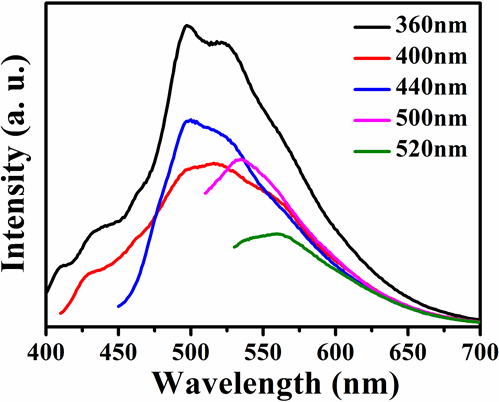


**Figure S2.** Fluorescence spectra of GQDs at different excitation wavelengths.
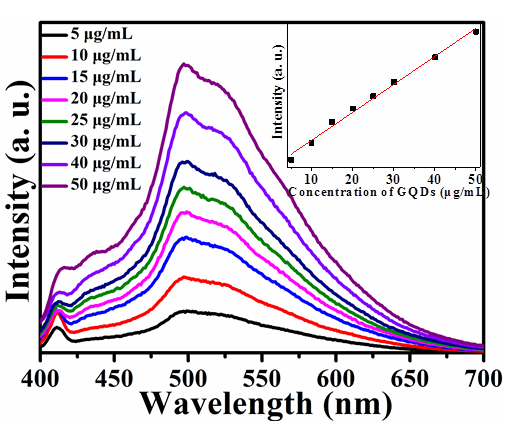


**Figure S3.** Concentration dependent PL spectrum of GQDs. The PL intensity of GQDs shows linear positive correlation with the concentration of GQDs in the range of 5-50 μM.





**Figure S4.** Inhibition effect of GQDs on the Aβ_1-42_ aggregation monitored by ThT assay.
